# Tuning properties and dynamic range of type 1 vomeronasal receptors

**DOI:** 10.3389/fnins.2015.00244

**Published:** 2015-07-14

**Authors:** Sachiko Haga-Yamanaka, Limei Ma, C. Ron Yu

**Affiliations:** ^1^Stowers Institute for Medical ResearchKansas City, MO, USA; ^2^Department of Anatomy and Cell Biology, University of Kansas Medical CenterKansas City, KS, USA

**Keywords:** vomeronasal receptor, calcium imaging, sulfated steroids, transgenic mice, glucuronidated steroid, GCaMP2

## Abstract

The mouse vomeronasal organ (VNO) expresses chemosensory receptors that detect intra-species as well as inter-species cues. The vomeronasal neurons are thought to be highly selective in their responses. The tuning properties of individual receptors remain difficult to characterize due to the lack of a robust heterologous expression system. Here, we take a transgenic approach to ectopically express two type 1 vomeronasal receptors in the mouse VNO and characterize their responses to steroid compounds. We find that V1rj2 and V1rj3 are sensitive to two sulfated estrogens (SEs) and can be activated by a broad variety of sulfated and glucuronidated steroids at high concentrations. Individual neurons exhibit narrow range of concentration-dependent activation. Collectively, a neuronal population expressing the same receptor covers a wide dynamic range in their responses to SEs. These properties recapitulate the response profiles of endogenous neurons to SEs.

## Introduction

Terrestrial animals emit pheromones for intra-species chemo-communication. Within the same species, pheromones convey information about sexual, social, and reproductive status of individuals. They trigger a restricted repertoire of innate and stereotyped behaviors, including mating rituals, territorial aggression, and neuroendocrine responses (Wyatt, 2003). The vomeronasal organ (VNO) plays an important role for detecting pheromones in most terrestrial vertebrate species (Halpern and Martinez-Marcos, 2003; Swaney and Keverne, 2009). The mouse VNO expresses about 400 seven-transmembrane G protein-coupled receptors including the type 1 vomeronasal receptors (V1Rs), type 2 vomeronasal receptors (V2Rs), and formyl peptide receptors (Dulac and Axel, 1995; Herrada and Dulac, 1997; Matsunami and Buck, 1997; Ryba and Tirindelli, 1997; Young and Trask, 2007; Liberles et al., 2009; Riviere et al., 2009; Young et al., 2010). Each vomeronasal sensory neuron (VSN) expresses either a single member of V1R or FPR, or a specific pair of V2R genes (Ibarra-Soria et al., 2014; Liberles, 2014). These receptors are dedicated to detect a variety of chemosensory cues and transmit the signal to the brain.

In natural environment, pheromone cues are present in bodily secretion and excretion, such as tears, feces, and urine (Ihara et al., 2013). The chemical nature of the pheromones ranges from volatile and non-volatile small molecular weight compounds to peptides and proteins (Novotny et al., 1985, 1986; Jemiolo et al., 1989; Leinders-Zufall et al., 2004; Kimoto et al., 2005; Chamero et al., 2007; Nodari et al., 2008; Riviere et al., 2009; Haga et al., 2010; Roberts et al., 2010, 2012; Ferrero et al., 2013). A unique pumping mechanism is required to bring the pheromone cues into the vomeronasal cavity where they are in contact with the dendrites of VSNs (Meredith et al., 1980; Meredith, 1994). In addition to their chemical identities, pheromone concentrations also convey specific information (He et al., 2010; Ihara et al., 2013). The vomeronasal system is able to detect cues at various concentrations and convey concentration-invariant signals to the brain (Arnson and Holy, 2013).

VSNs are able to detect pheromone cues with high sensitivity and selectivity. In some studies, VSNs can respond specifically to ligands at sub-nanomolar concentrations (Leinders-Zufall et al., 2000, 2004, 2014; Kimoto et al., 2005; He et al., 2010; Isogai et al., 2011; Haga-Yamanaka et al., 2014). It is not understood how the VSN achieve a balance between receptor sensitivity and a wide dynamic range of detection. There are two plausible mechanisms that can be considered. In one scenario, neurons expressing the same receptor may exhibit different sensitivities to accommodate a wide range of ligand concentrations. Alternatively, neurons expressing different receptors with distinct sensitivities to the ligand broadens the detectable range of pheromones. Current available data do not distinguish these two hypotheses.

Thus, in order to understand the mechanisms of pheromone tuning, it is important to characterize the specific interactions between pheromones and their cognate receptors. However, after nearly 20 years of cloning of the first V1Rs, we have limited understanding of the receptor characteristics and functions. The main obstacle is the lack of a robust heterologous expression system that enables functional analysis of VR responses. In an earlier study, we overcome this challenge by generating transgenic mice that ectopically express V1Rs in VSNs (Haga-Yamanaka et al., 2014). The ectopic expression of vomeronasal receptors in their native environment enables us to identify V1rj2 and V1rj3 receptors as the cognate receptors for sulfated estrogens (SEs). In the current study, we further characterize the response profiles of V1rj2 and V1rj3 receptors to a panel of sulfated and glucuronidated steroids.

## Materials and methods

### Mice

All mice were maintained in Lab Animal Service Facility of Stowers Institute at 12:12 light/dark cycle and provided with food and water *ad libitum*. Experimental protocols were approved by the Institutional Animal Care and Use Committee at Stowers Institute and in compliance with NIH Guide for Care and Use of Animals. OMP-IRES-tTA (OIVT), Gγ8-tTA, tetO-GCaMP2, tetO-V1rj2-IRES-tdTomato, and tetO-V1rj3-IRES-tdTomato mice were described previously (Yu et al., 2004; Nguyen et al., 2007; He et al., 2008; Haga-Yamanaka et al., 2014). Mice containing OIVT, Gγ8-tTA, and tetO-GCaMP2 alleles were used to assess the wild type responses. Compound heterozygotic mice that contained tetO-V1rj2-IRES-tdTomato and tetO-V1rj3-IRES-tdTomato were generated to assess the V1rj2 and V1rj3 responses, respectively. VNO slices were prepared from 2 to 6 months old male and female mice.

### Chemicals

Sulfated and glucuronidated steroids were purchased from Steraloids (Newport, RI, USA), and the catalog IDs were used to label the compounds. We dissolved the steroids in dimethyl sulfoxide (DMSO) to make 20 mM stock solutions, which were further diluted in Ringer's solution (in mM: 125 NaCl, 2.5 KCl, 2 CaCl_2_, 2 MgCl_2_, 25 NaHCO_3_, 10 HEPES, and 10 glucose) to the testing concentrations.

### Calcium imaging of VNO slices

Details of imaging setup and procedures were described previously (Ma et al., 2011). Briefly, VNO slices were maintained in carboxygenated (95% O_2_, 5% CO_2_) mouse artificial cerebrospinal fluid (mACSF; in mM: 125 NaCl, 2.5 KCl, 1 MgCl_2_, 2 CaCl_2_, 1.25 NaH_2_PO_4_, 25 NaHCO_3_, and 10 glucose) at room temperature. Carboxygenated mACSF was also used to superfuse VNO slices at a speed of 1 mL/min. The flow was kept unidirectional by placing the inlet and outlet at the apical and basal sides of VNO epithelium, respectively. Steroids were delivered through a HPLC injection valve mounted on the stage. To minimize mechanical artifacts, a continuous flow (~0.3 mL/min) of Ringer's solution was maintained during the experiment. Solutions were switched by using the injection valve without disrupting the flow.

Time-lapse imaging acquisition of GCaMP2 signals from a VNO slice was performed on AxioScope FS2 (Carl Zeiss) microscope with a 20X/0.5NA water-dipping lens as described before (He et al., 2008; Ma et al., 2011; Yu, 2013; Haga-Yamanaka et al., 2014). Intervals between stimuli were at least 3 min. When a robust response to a given chemical was observed, we set the inter-stimulus interval to 5–10 min (varied with response level) to ensure adequate recovery. Data of individual genotypes or applications were collected from two to three VNO slices. Image processing and data analysis, including region of interest (ROI) detection and automated signal analyses, were performed using ImageJ and programs written in MATLAB. In order to compute F, a baseline fitting step was performed to model photo-bleaching effect and a peak detection step was performed to model the signal peak. ΔF was computed as the difference between the signal peak and the baseline. The computation was then manually validated to exclude possible errors. A threshold of 30% ΔF/F was imposed to identify VSNs as responding to a stimulus. Response amplitudes were normalized in each individual cell. For ensemble responses, the dose-response curve was fitted with a sigmoidal equation and EC50 were calculated using Prism (GraphPad).

## Results

### VSN response to sulfated estrogens

**Figure 1:**
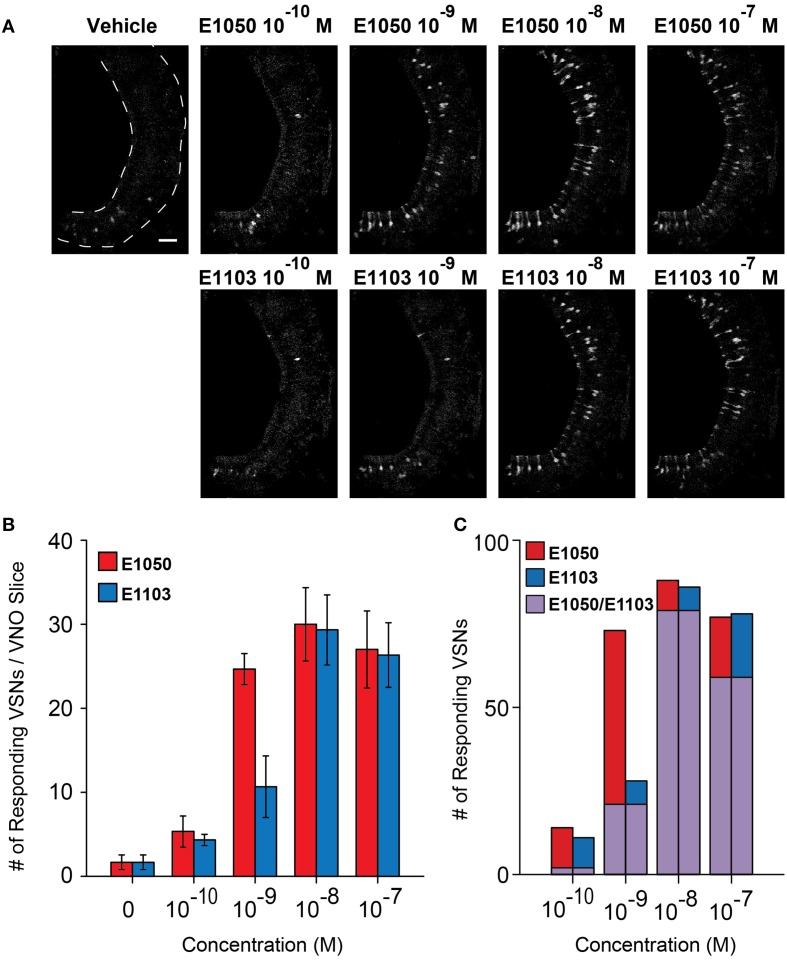
**Activation of VSN by E1050 and E1103. (A)** Representative imaging experiments showing the VSN responses to E1050 (top) and E1103 (bottom). Scale bar, 50 μm. **(B)** Bar graph showing the number of VSNs activated by E1050 (red) and E1103 per slice (blue; *n* = 3 slices). Error bars, S.E.M. **(C)** Bar graph showing the number of E1050- and/or E1103-responding VSNs in 3 slices. Red, blue, and purple indicate VSNs activated by E1050, E1103, and both E1050 and E1103, respectively.

Two of the SEs, 1, 3, 5(10)-estratrien-3, 17β-diol disulfate (E1050), and 1, 3, 5(10)-estratrien-3, 17β-diol 17-sulfate (E1103), strongly activate VSNs and can serve as estrus signals to induce mounting behaviors in male mice (Haga-Yamanaka et al., 2014). To understand the dose-dependent activation of VSNs, we performed Ca^2+^ imaging experiments using VNO slices from GCaMP2 mice. We observed VSN responses to E1050 and E1103 at 10^−10^ M in ~2–4% of cells on a single slice (Figures 1A,B). The number of responding VSNs increased in a dose-dependent manner and reached a plateau of approximately 30 cells per slice, which represented approximately 15% of the cell population (Figures 1A,B).

**Figure 2:**
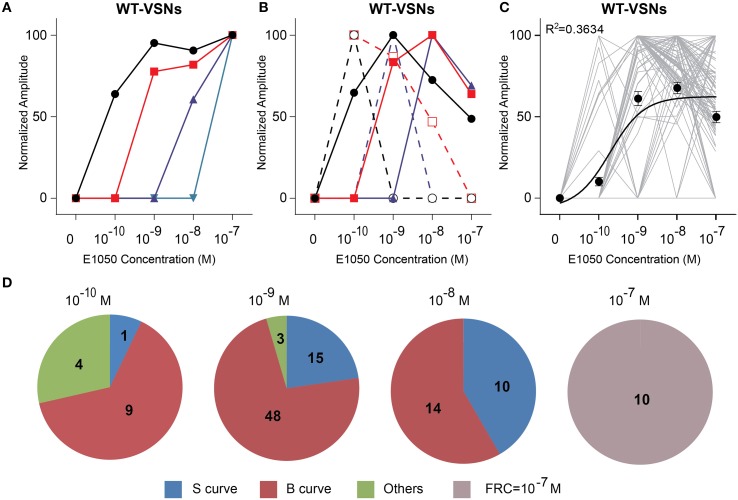
**Activation of VSN by E1050. (A,B)** Examples of sigmoidal **(A)** and bell-shaped **(B)** dose-response curves to E1050. **(C)** Dose-response curves of individual cells (gray) and a sigmoidal curve (black) fitted to the average amplitude from all cells. Error bar, S.E.M. **(D)** Pie charts showing the number of cells exhibiting sigmoidal (S, blue), bell-shaped (B, red), and other (O, green) types of dose responses. Cells with FRC of 10^−7^ M are shown in gray. Concentration above each pie chart represents the first response concentration of the cells.

A comprehensive survey of the VSN response profiles showed that individual neurons exhibited diverse dose-response properties and sensitivities to E1050. Some cells showed the classic sigmoidal dose-response curves with increasing amplitude as a function of E1050 concentration (Figures 2A,C,D). The signal plateaued at higher concentrations, which indicated a saturation of the response. Neurons exhibiting these classic dose-response curves, however, only represented a fraction of the total VSNs. We found a large fraction (~60%) of neurons displayed bell-shaped curves (Figures 2B–D). The peak response was reached at an intermediate concentration. Further increase of ligand concentration led to reduced response. A few cells displayed dose-response properties that did not fit either sigmoidal or bell-shaped curves (Figures 2C,D).

We also observed VSNs displayed different sensitivities to SE activation (Figure 2D). We used the first response concentration (FRC) as a measurement of sensitivity. Overall, the FRCs varied at least four orders of magnitude from 10^−10^ to 10^−7^ M. We observed some cells started to respond at 10^−7^ M, which was the highest concentration tested for these two SEs. We marked those high-threshold cells as with FRC at 10^−7^ M. Regardless of the shape of their dose-response curves, individual neurons had relatively narrow dynamic ranges. Approximately 90% of neurons showed the maximal response at 10x FRC.

Although individual neurons had different sensitivities and narrow dynamic ranges, VSN population can respond to a wide range of pheromone stimulation collectively (Figure 2C). The average response to E1050 had an EC50 of 1.921×10^−10^ M with a dynamic range of 1000 fold change in concentration.

**Figure 3:**
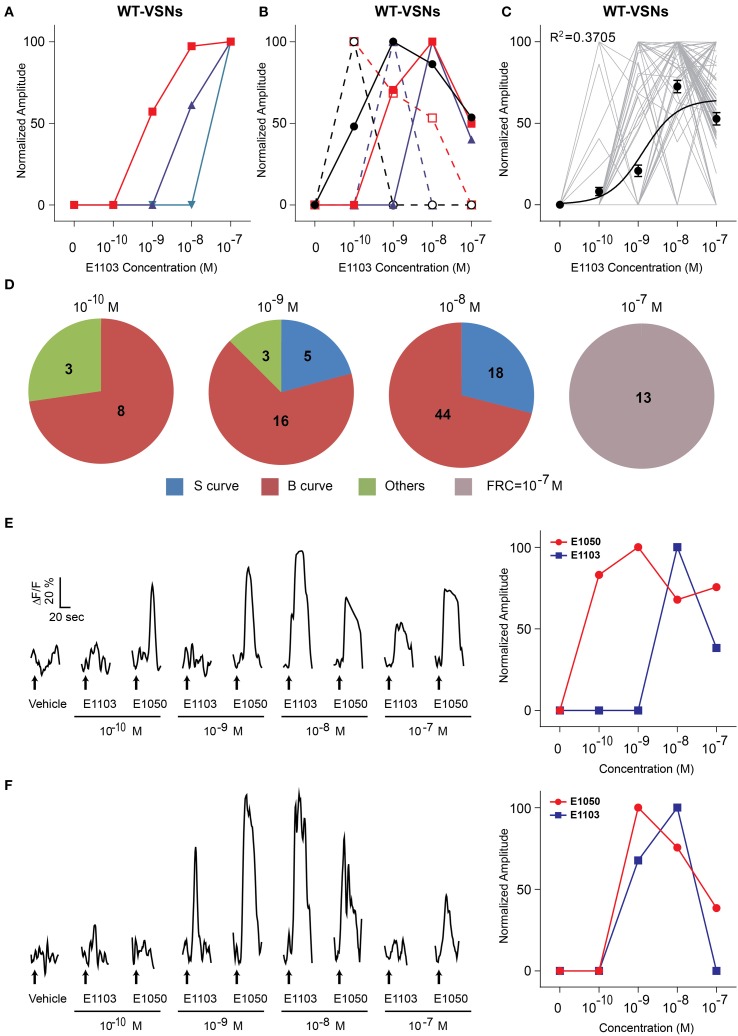
**Activation of VSN by E1103. (A,B)** Examples of sigmoidal **(A)** and bell-shaped **(B)** dose-response curves to E1103. **(C)** Dose-response curves of individual cells (gray) and a sigmoidal curve (black) fitted to the average amplitude from all cells. Error bar, S.E.M. **(D)** Pie charts showing the number of cells exhibiting sigmoidal (S, blue), bell-shaped (B, red) and other (O, green) types of dose responses. Cells with FRC of 10^−7^ M are shown in gray. **(E,F)** Raw traces (left) and dose-response curves (right) of two representative cells simulated by E1050 and E1103 in a sequence with increasing concentrations. Arrows indicate the onset of stimulus delivery.

The responses to E1103, a singly-sulfated estrogen compound, elicited VSN responses at as low as 10^−10^ M (Figures 1A,B). At this concentration, ~85% of the neurons activated by E1103 and E1050 were distinct (Figure 1C). Compared to E1050, the number of E1103 responding VSNs showed a slower increase with rising concentration and did not plateau until 10^−8^ M (Figure 1B). At concentrations higher than 10^−9^ M, the majority of E1103 responding VSNs overlapped with E1050 responding cells (Figure 1C). At individual cell level, the sensitivity ranged across three orders of magnitude. Consistent with the number of responding VSNs, we found that a large fraction of the cells showed peak response to E1103 at 10^−8^ M. Both sigmoidal and bell-shaped dose-response curves were observed (Figures 3A,B,D). On average, responses to E1103 had an EC50 of 1.348×10^−9^ M (Figure 3C).

The bell-shaped dose-response curve also has been observed in urine-responding VSN neurons (He et al., 2010). Because the VSNs showed little adaptation to repeated or prolonged stimuli (Holy et al., 2000; He et al., 2010), it was unlikely that the diminution of response at high ligand concentration was caused by cell fatigue or receptor desensitization. To further exclude these possibilities, we examined the response of individual cells to different ligands. As shown in Figures 3E,F, the same cell exhibited reduced response to high concentration (10^−7^ M) of E1103, but subsequent application of E1050 nonetheless elicited strong responses. The same cells, therefore, exhibited distinct dose-dependent activations to different ligands that was not related to stimulation sequence.

### Activation of V1rj2 by sulfated estrogens

In a previous study, we showed that the V1rj clade receptors respond to SEs by using transgenic mice lines in which V1rj2 and V1rj3 were ectopically expressed (Haga-Yamanaka et al., 2014). These mice allowed us to further examine the activation of individual V1rj receptors activated by SEs.

**Figure 4:**
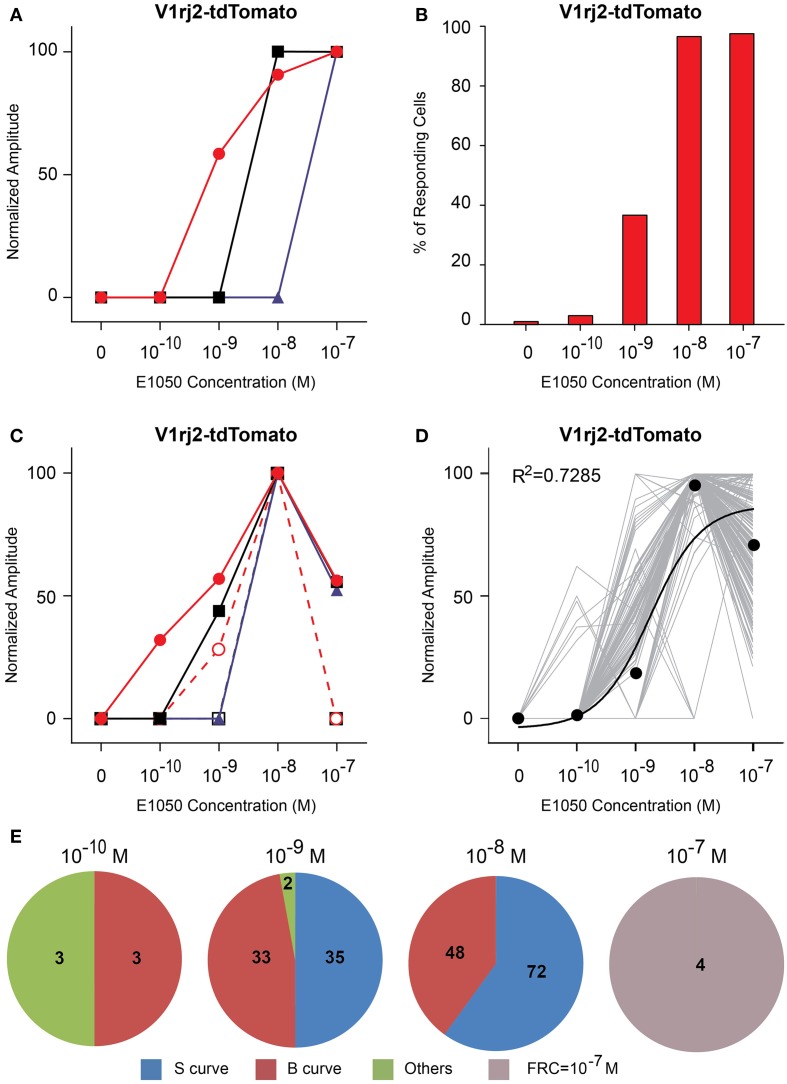
**Activation of V1rj2 by E1050. (A)** Examples of sigmoidal dose-response curves to E1050 in V1rj2 cells. **(B)** Bar graph showing the percentage of E1050 activated V1rj2 cells (*n* = 200). **(C)** Examples of bell-shaped dose- response curves to E1050 in V1rj2 cells. **(D)** Dose-response curves of individual cells (gray) and a sigmoidal curve (black) fitted to the average amplitude from all cells. Error bar, S.E.M. **(E)** Pie charts showing the number of cells exhibiting sigmoidal (S, blue), bell-shaped (B, red), and other (O, green) types of dose responses. Cells with FRC of 10^−7^ M are shown in gray.

**Figure 5:**
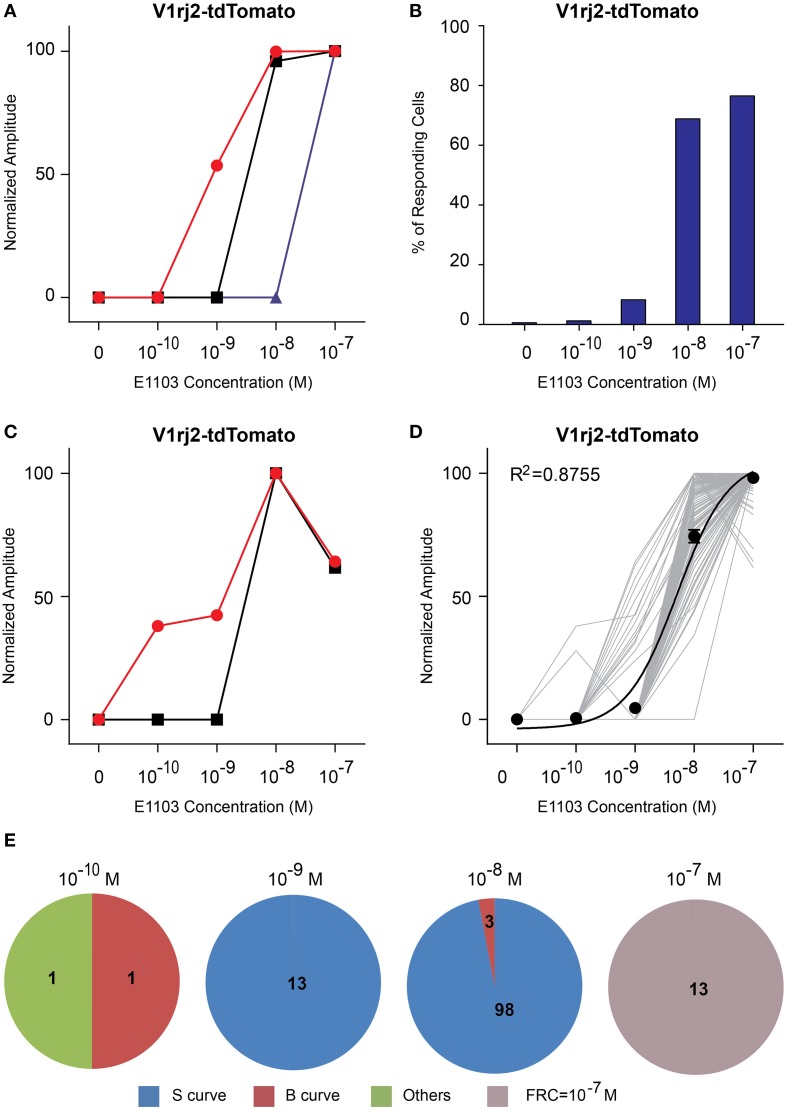
**Activation of V1rj2 by E1103. (A)** Examples of sigmoidal dose-response curves to E1103 in V1rj2 cells. **(B)** Bar graph showing the percentage of E1103 activated V1rj2 cells (*n* = 129). **(C)** Examples of bell-shaped dose-dependent responses of V1rj2 cells to E1103. **(D)** Dose-response curves of individual cells (gray) and a sigmoidal curve (black) fitted to the average amplitude from all cells. Error bar, S.E.M. **(E)** Pie charts showing the number of cells exhibiting sigmoidal (S, blue), bell-shaped (B, red), and other (O, green) types of dose responses. Cells with FRC of 10^−7^ M are shown in gray.

V1rj2-expressing VSNs showed dose-dependent activation by both SEs (Figures 4, 5). E1050 activated V1rj2-expressing VSNs at 10^−10^ M and the numbers of responding cells plateaued at 10^−8^ M (Figure 4B). These VSNs showed both the classic dose-response curves and bell-shaped curves (Figures 4A,C,E). In addition, the majority of V1rj2-expressing VSNs started responding to E1050 at 10^−9^ M and reached the maximal response amplitude at 10^−8^ M (Figure 4D). On average, the V1rj2 neurons covered a dynamic range of 1000 folds (Figure 4D).

Compared to E1050, fewer V1rj2-expressing VSNs were activated by E1103 at 10^−10^ M (Figure 5B). Interestingly, the majority of VSNs exhibited classic dose-response curves (Figure 5E). Most of V1rj2-expressing VSNs started to respond at 10^−8^ M and peaked at 10^−7^ M (Figures 5B,D).

### Activation of V1rj3 by sulfated estrogens

**Figure 6:**
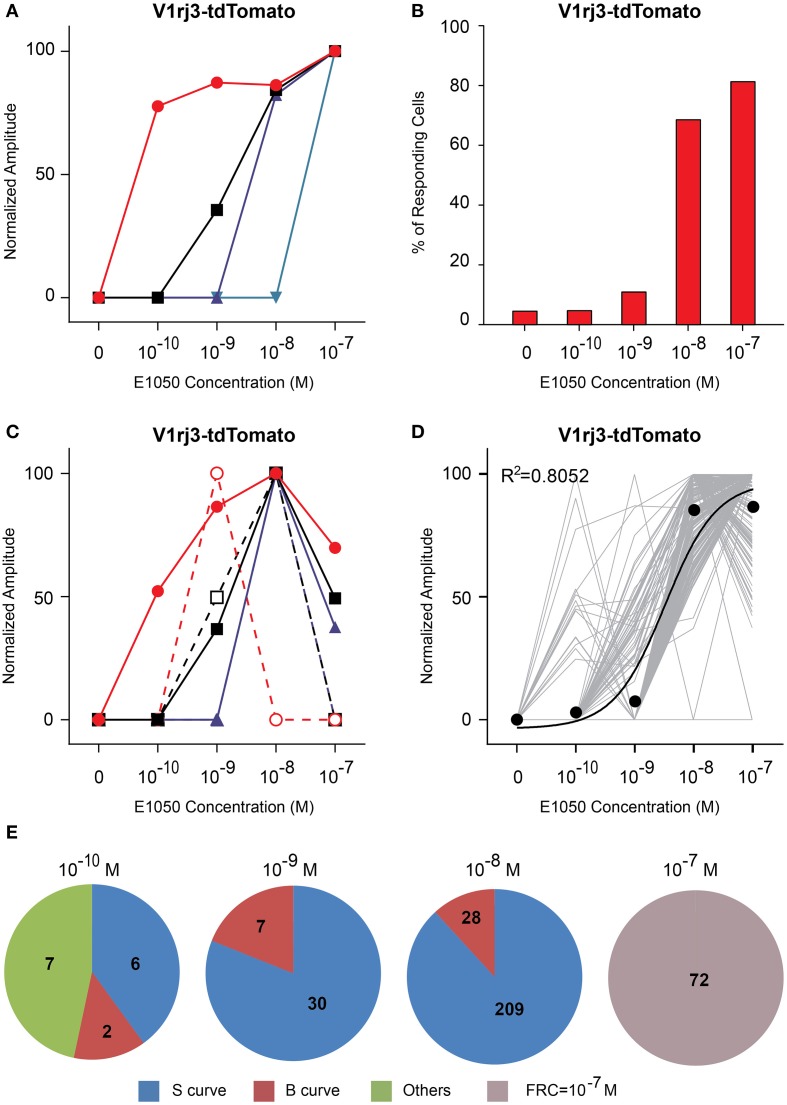
**Activation of V1rj3 by E1050. (A)** Examples of sigmoidal dose-response curves to E1050 in V1rj3 cells. **(B)** Bar graph showing the percentage of E1050 activated V1rj3 cells (*n* = 256). **(C)** Examples of bell-shaped dose-dependent responses of V1rj3 cells to E1050. **(D)** Dose-response curves of individual cells (gray) and a sigmoidal curve (black) fitted to the average amplitude from all cells. Error bar, S.E.M. **(E)** Pie charts showing the number of cells exhibiting sigmoidal (S, blue), bell-shaped (B, red), and other (O, green) types of dose responses. Cells with FRC of 10^−7^ M are shown in gray.

Compared to V1rj2 cells, VSNs expressing the V1rj3 receptor showed distinct response characteristics to E1050 (Figure 6). The majority of the cells only responded at or above 10^−8^ M (Figure 6B) and less than 30% of the cells exhibited bell-shaped response curves (Figure 6E). A significant number of V1rj3-expressing cells were activated by E1050 at 10^−10^ M and showed maximal response at this concentration (Figure 6D). These populations of VSNs make this single receptor cover a wide dynamic range in their response to SEs (Figure 6D).

**Figure 7:**
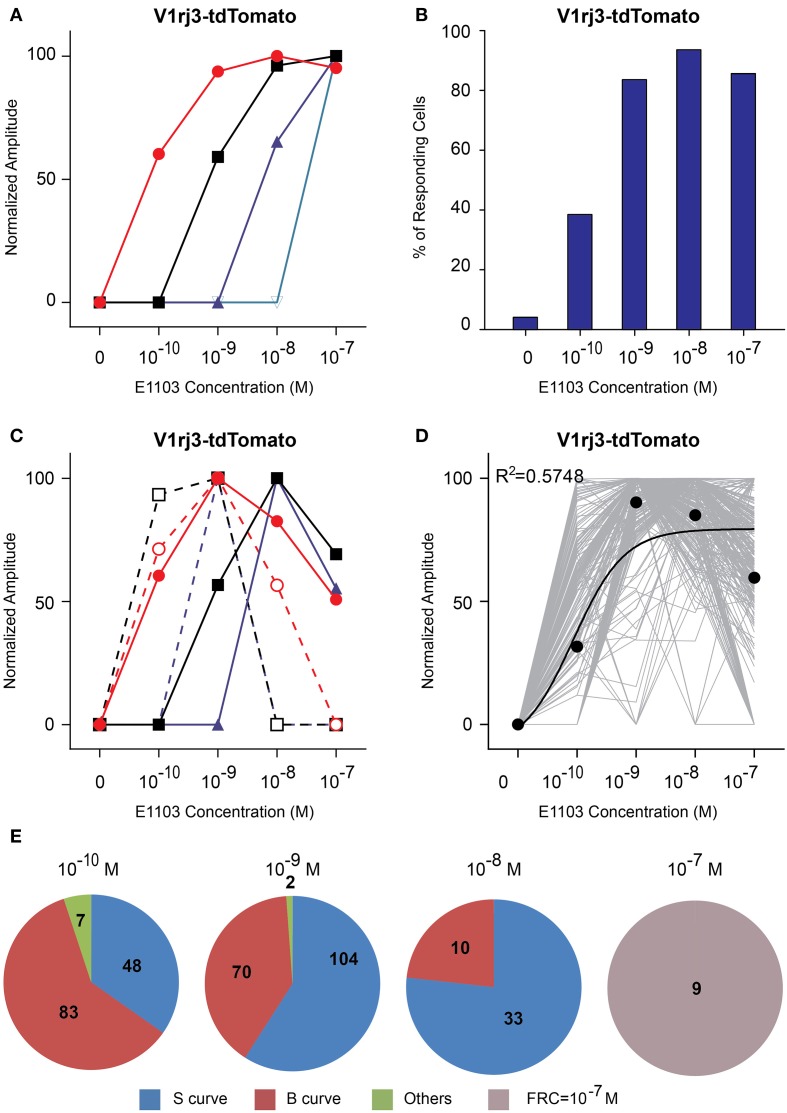
**Activation of V1rj3 by E1103. (A)** Examples of sigmoidal dose-response curves to E1103 in V1rj3 cells. **(B)** Bar graph showing the percentage of E1103 activated V1rj3 cells (*n* = 256). **(C)** Examples of bell-shaped dose-dependent responses of V1rj3 cells to E1103. **(D)** Dose-response curves of individual cells (gray) and a sigmoidal curve (black) fitted to the average amplitude from all cells. Error bar, S.E.M. **(E)** Pie charts showing the number of cells exhibiting sigmoidal (S, blue), bell-shaped (B, red), and other (O, green) types of dose responses. Cells with FRC of 10^−7^ M are shown in gray.

The V1rj3-expressing cells also responded to E1103 with high sensitivities (Figure 7). About 50% of the cells responded at 10^−10^ M (Figure 7B). The number of responding cells plateaued at 10^−8^ M. Approximately 45% of the cells exhibited bell-shaped dose-response curves, with the maximal responses elicited between 10^−10^ and 10^−8^ M (Figures 7D,E).

These results suggested that the V1rj2 receptor was more sensitive to E1050, while the V1rj3 receptor was more sensitive to E1103. At the individual cell level, both V1rj2 and V1rj3 VSNs exhibited rather narrow dynamic range. The maximal response was typically observed at a concentration just 10x of FRC. Collectively, each receptor type was able to cover the concentration range of SE spanning three orders of magnitude.

### Activation of V1rj2 receptor by glucuronidated estrogens

Steroid hormones can be modified after initial synthesis and during circulation. Circulating estrogen molecules are modified by specific enzymes to become sulfated or glucuronidated estrogens (GEs), which are soluble and can be excreted in urine (Shackleton, 1986; Blair, 2010). We therefore examined whether GEs also activated the V1rj2 and V1rj3 receptors using mono-glucuronidated estrogen molecules 1, 3, 5(10)-estratrien- 3, 17β-diol 3-glucosiduronate (E1072) and 1, 3, 5(10)-estratrien-3, 17β- diol 17-glucosiduronate (E1073). The 3- and 17-hydroxyl groups are glucuronidated in E1072 and E1073, respectively.

**Figure 8:**
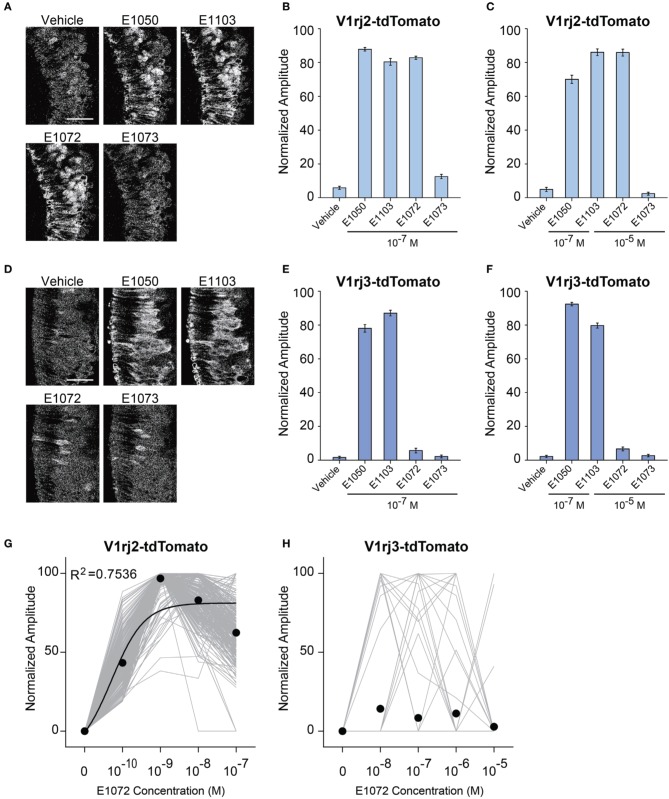
**Activation of V1rj2 and V1rj3 by Glucuronidated Estrogens. (A)** Representative imaging experiments showing the responses of V1rj2 cells to vehicle control, E1050, E1103, E1072, and E1073 at 10^−7^ M. Scale bar, 50 μm. **(B)** Bar graph showing the normalized response to E1050, E1103, E1072, and E1073 in V1rj2 cells at indicated concentration (*n* = 409). **(C)** Bar graph showing the normalized response to E1050, E1103, E1072, and E1073 in V1rj2 cells at indicated concentrations (*n* = 409). **(D)** Representative imaging experiments showing the responses of V1rj3 cells to vehicle control, E1050, E1103, E1072, and E1073 at 10^−7^ M. **(E)** Bar graph showing the normalized response to E1050, E1103, E1072, and E1073 in V1rj3 cells at indicated concentration (*n* = 204). **(F)** Bar graph showing the normalized response to E1050, E1103, E1072, and E1073 in V1rj3 cells at indicated concentrations (*n* = 318). **(G,H)** Dose-response curve of V1rj2 **(G)** and V1rj3 **(H)** cells activated by E1072. Error bars, S.E.M.

At 10^−7^ M, E1072 stimulated responses in V1rj2 cells (Figures 8A,B). The response amplitude was comparable to that activated by E1050 and E1103 at the same concentration. On the other hand, E1073 did not induce any response in V1rj2 expressing VSNs at concentrations up to 10^−5^ M (Figures 8A–C). Dose-response analysis indicated that V1rj2 cells were highly sensitive to E1072. Neurons were activated at 10^−10^ M and reached the maximal response at 10^−9^ M (Figure 8G). These cells also showed narrow dynamic ranges. Similar to the SEs, we observed bell-shaped dose-response curves that peaked at concentrations between 10^−9^ and 10^−7^ M. In contrast, V1rj3-expressing VSNs did not respond to E1072 or E1073 more than the vehicle controls (Figures 8D–F,H). These results demonstrated that E1072 was a ligand for V1rj2 and E1073 could not activate either receptors.

### Responses of V1rj2 and V1rj3 to other sulfated steroids

**Figure 9:**
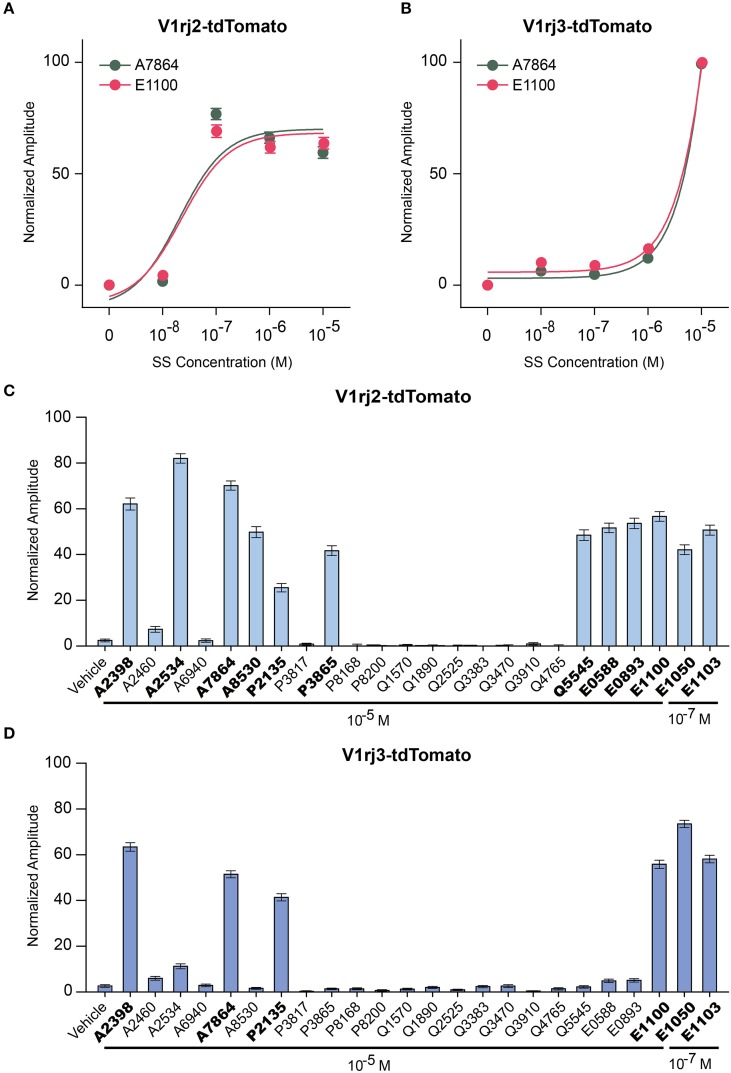
**Activation of V1rj2 and V1rj3 by Other Sulfated Steroids. (A)** Dose-response curves of V1rj2 cells activated by A7864 (*n* = 201) and E1100 (*n* = 199). **(B)** Dose-response curves of V1rj3 cells activated by A7864 (*n* = 249) and E1100 (*n* = 249). **(C,D)** Bar graphs showing the normalized response of V1rj2 (**C**; *n* = 183) and V1rj3 (**D**; *n* = 373) cells a panel of sulfated steroid compounds. Error bars, S.E.M.

Previous studies have shown that individual VSNs are activated by different steroid compounds (Nodari et al., 2008; Meeks et al., 2010; Isogai et al., 2011). We also observed that V1rj2 expressing VSNs responded to a sulfated androgen, 5-androsten-3β, 17β-diol-disulfate (A7864), and other members of SEs, such as 17β-estradiol 3-sulfate (E1100) (Haga-Yamanaka et al., 2014). Here, we analyzed the response of V1rj2 and V1rj3 to these two compounds. Approximately 80% of V1rj2 expressing VSNs began to respond to both A7864 and E1100 at 10^−7^ M. The response amplitude plateaued at 10^−7^ M and increasing ligand concentration did not further augment the response amplitude (Figure 9A). A7864 and E1100 were able to activate V1rj3 at 10^−5^ M (Figure 9B).

To obtain a comprehensive understanding of ligand selectivity of these V1R receptors, we tested a panel of sulfated steroids at 10^−5^ M (Figures 9C,D). V1rj2 expressing VSNs were activated by multiple sulfated androgens including A2398, A2534, A7864, and A8530. They also responded to a couple of sulfated progesterones including P2135 and P3865, a corticosterone, Q5545, and all the SEs tested including E0588, E0893, and E1100 (Figure 9C). V1rj3 cells were more selective in their responses. They were activated by a couple of sulfated androgens, A2398 and A7864, sulfated progesterone, P2135, and one SE, E1100 (Figure 9D). V1rj3 cell did not respond to sulfated corticosterone stimulation. Thus, V1rj2 and V1rj3 receptors were activated by a diverse group of sulfated steroids and showed tuning preference to different compounds.

### Structural basis of V1rj2 and V1rj3 activation

**Figure 10:**
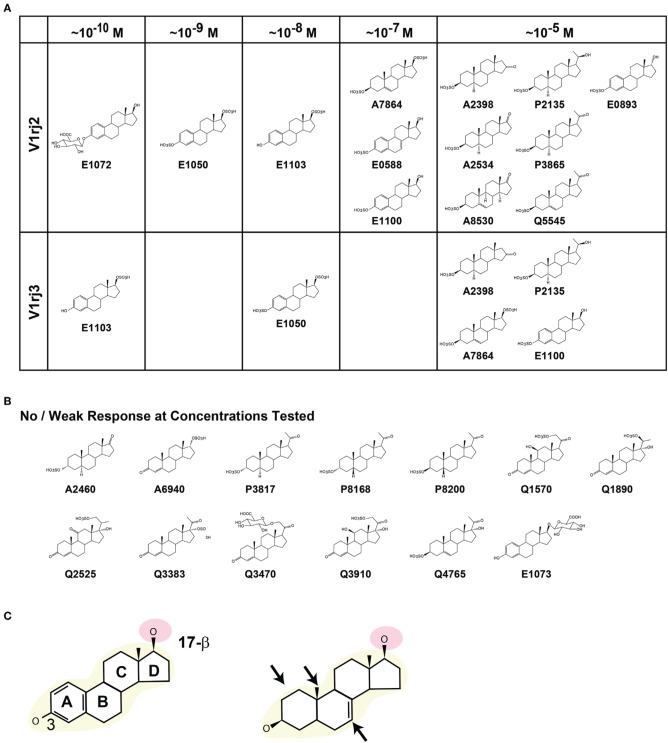
**Structural elements of steroid compounds that activate V1rj2 and V1rj3 receptors. (A)** Steroid compounds that activate V1rj2 and V1rj3 receptors. The minimal concentrations of activation are indicated. **(B)** Molecular structures of sulfated or glucuronidated steroids that induce no or weak responses. **(C)** Schematic illustration of important structural elements required to activate the V1rj2 and V1rj3 receptors. The estradiol backbone (yellow) and 17-β hydroxyl group (pink) are highlighted. Structural permutations that are permissible are indicated by arrows in the right panel.

The two V1rj receptors share 81% amino acid sequence similarity but exhibit differential tuning and dose-response relationship to steroid derivatives. By comparing the molecular structures of the sulfated and glucuronidated steroids, it appears that a core estradiol-17β structure is essential for highly sensitive activation of the receptors (Figure 10). The 3- and 17β-hydroxyl groups appear to be important structural determinants. The steroid compounds maintain high potency when the 3-hydroxyl group is unmodified, sulfated or glucuronidated. However, compounds with a ketone group at this position, such as A6940, lose their ability to activate the receptors. The β configuration of the 17-hydroxyl group is important, too. E0893, which has α configuration, is two orders of magnitude less potent in activating the V1rj2 receptor than E0588 and E1100. This observation suggests that the configuration at the 17-β site plays an important role in activating the receptors. Glucuronidation at this site, as in E1073, blocks its activity, whereas the native or sulfated form retains activity.

We observe that the most potent compounds possess an unsaturated A ring, but permutations in this structure appear to be tolerated (Figure 10C). Compounds that possess unsaturated A ring, 18-methyl group or unsaturated bonds in the B ring activate the receptors as long as the 3 and 17β-hydroxyl groups are intact.

## Discussion

By taking advantage of a transgenic system to ectopically express the V1Rs, we circumvent a major obstacle in characterizing vomeronasal receptors. Although the VRs can be expressed in heterologous systems, the receptors are retained in the endoplasmic reticulum and cannot be transported to the cell surface. Chaperone proteins, including receptor-transporting proteins (RTPs) and receptor expression enhancing proteins (REEPs), have been shown to facilitate transportation of some odorant receptors to the cell membrane (Saito et al., 2004; Mainland and Matsunami, 2012). None of these molecules can facilitate the surface expression of V1Rs. Although several molecules have been reported to be involved in VR surface expression, it is still challenging to examine individual VR function in heterologous expression systems (Ishii et al., 2003; Loconto et al., 2003; Dey and Matsunami, 2011). In our transgenic system, individual VRs can be ectopically expressed via a synthetic tetO promoter and transported to the cell surface utilizing the endogenous chaperons in VSNs. Thus, this system allows us to characterize VR response in its native environment.

### Recapitulation of native responses

Previously we observed a diverse responses of VSNs to urinary pheromone stimulation (He et al., 2008, 2010). It was not clear whether the variations in responses arose from the mixture of pheromone cues in the urine or from diverse properties of the VSNs. In the transgenic lines, we also observe that the VSNs display a variety of dose-dependent activation by individual pheromone ligands. Sensitivities of individual cells to SEs range from sub-nanomolar to -micromolar concentrations. The overall dynamic range of the response of the two receptors are comparable to that of the endogenous receptors to SE ligands.

Individual V1rj2 or V1rj3 expressing neurons show both sigmoidal and bell-shaped dose-response curves, similar to those observed in wild type VSNs. Different dose-response relationships appear to be specific to individual cells. We notice that a smaller percentage of V1rj2 and V1rj3 cells exhibit bell-shaped dose-response curves than the wild type VSNs. The difference may reflect the level of expression of receptor proteins in the transgenic mice, which may affect the coupling between receptor and downstream signaling pathways. Alternatively, the dose-response properties may be intrinsic to individual cells. As the V1rj receptors are expressed in most of the VSNs in the transgenic line, the difference in cellular properties may affect how the ectopically expressed receptors respond to ligands.

The cellular mechanism that generates the bell-shaped dose-response curves is unknown. Experimental evidence from multiple studies suggests that they are unlikely the result of receptor desensitization or cell fatigue. Calcium signal is usually correlated with spiking activity. When a cell is stimulated by a high concentration of ligand, it is possible that a large depolarization inactivates voltage-gated sodium and calcium channels such that the cell cannot sustain action potentials and Ca^2+^ entry through the Ca^2+^ channels. As voltage-gated channels can recover quickly from refractory period, this scenario could explain why the cells can respond to different ligands differently at high concentrations. Our result puts the focus on this phenomenon, and further analyses using electrophysiological recording could reveal the cellular mechanism.

### Sensitivity and dynamic range of the VSNs

The ectopically expressed V1rj receptors are activated by SEs at concentrations as low as 10^−10^ M. It appears that more V1rj3 cells respond to the ligands at this low concentration than V1rj2 cells. The majority of the cells show response saturation within two orders of magnitude. An efficient intracellular signal amplification process is likely to couple the response. The VNO has unusual signal transduction mechanisms (Kim et al., 2012). Different signaling cascades could allow low concentration of ligands to elicit a neuronal response to achieve heightened sensitivity. An amplification process may also explain the relatively narrow dynamic range at individual cell level. A relatively moderate increase in ligand concentration may quickly saturate the responses. In some cells, overstimulation may cause desensitization, which can result in a decline of responses as shown in the bell-shaped dose-response curves to steroid compounds and to mouse urine (He et al., 2010).

VSNs expressing the same receptor exhibit different sensitivities. Since each cell also display sharp changes in dose-dependent activation, the sensitivity is unlikely to reflect differences in intrinsic amplification. It is more likely that sensitivity is determined by the amount of receptors expressed on the cell surface. Alternatively, the binding affinity of the VNO receptors might be affected by the membrane environment in which they are expressed. Our current study, however, does not have evidence to fully address this question.

Despite the narrow dynamic range of individual cells, the VSN population respond to a wide range of ligand concentrations. The average dose-response curve spans at least three orders of magnitude. This allows the VNO to accommodate a large quantitative variation of pheromones presented by animals. VSNs expressing the same vomeronasal receptor project stereotypically to multiple glomeruli in the accessory olfactory bulb (Belluscio et al., 1999; Rodriguez et al., 1999; Wagner et al., 2006; Haga et al., 2010; Ishii and Mombaerts, 2011). Each glomerulus likely receives input from VSNs expressing the same VR. This convergence pattern may allow each glomerulus to integrate signals transmitted by neurons with disparate sensitivity such that the glomerulus serves as a proxy of the VSN collectives. Alternatively, it is also possible that VSN axons segregate in their projection patterns according to ligand sensitivity such that each of the multiple glomeruli exhibits narrow concentration tuning. This scenario may provide a mechanism to readout concentration ratios for concentration-invariant perception of the pheromone cues. Detailed study of the glomerular responses in the AOB would distinguish these two models.

Our study also shows that neurons expressing the same receptor could be tuned to different steroid compounds at high concentrations. Therefore, in addition to chemical identities of ligands, concentration information about a ligand could also be transmitted by different receptors.

### Tuning specificity and steric configuration of the molecules

V1rj2 and V1rj3 are sensitive to SE compounds. They are also activated to a lesser extent by other sulfated and glucuronidated steroids. The structural determinants of the activation appear to require the 3- and 17-hydroxyl groups and estradiol backbone. V1rj3 appears to be more sensitive to SEs, but are also tuned to fewer compounds than V1rj2. The specific interaction of these structural elements with the receptors may determine the specificity and sensitivity of V1rj2 and V1rj3 receptors. The structure basis that differentiates these two receptors remains unknown.

### Conflict of interest statement

The authors declare that the research was conducted in the absence of any commercial or financial relationships that could be construed as a potential conflict of interest.
